# EDTP enhances and protects the fluorescent signal of GFP in cleared and expanded tissues

**DOI:** 10.1038/s41598-024-66398-y

**Published:** 2024-07-03

**Authors:** Ruili Feng, Jiongfang Xie, Liang Gao

**Affiliations:** 1 Fudan University, Shanghai, 200433 China; 2 Key Laboratory of Structural Biology of Zhejiang Province, School of Life Sciences, Westlake University, Hangzhou, 310024 Zhejiang China; 3 Westlake Laboratory of Life Sciences and Biomedicine, Hangzhou, 310024 Zhejiang China

**Keywords:** Tissue clearing, Tissue expansion, EDTP, Tiling light sheet microscopy, Biochemistry, Biological techniques, Biotechnology

## Abstract

Advanced 3D high-resolution imaging techniques are essential for investigating biological challenges, such as neural circuit analysis and tumor microenvironment in intact tissues. However, the fluorescence signal emitted by endogenous fluorescent proteins in cleared or expanded biological samples gradually diminishes with repeated irradiation and prolonged imaging, compromising its ability to accurately depict the underlying scientific problem. We have developed a strategy to preserve fluorescence in cleared and expanded tissue samples during prolonged high-resolution three-dimensional imaging. We evaluated various compounds at different concentrations to determine their ability to enhance fluorescence intensity and resistance to photobleaching while maintaining the structural integrity of the tissue. Specifically, we investigated the impact of EDTP utilization on GFP, as it has been observed to significantly improve fluorescence intensity, resistance to photobleaching, and maintain fluorescence during extended room temperature storage. This breakthrough will facilitate extended hydrophilic and hydrogel-based clearing and expansion methods for achieving long-term high-resolution 3D imaging of cleared biological tissues by effectively safeguarding fluorescent proteins within the tissue.

## Introduction

Connectome mapping is pivotal for the analysis of neural networks, encompassing the spatial distribution of neurons in neural tissue, dendritic morphology, axonal projections, and structural information regarding synaptic connections. Due to the inherent opacity of biological tissues, conventional methods rely on slicing, imaging, and image reconstruction techniques to observe their high-resolution 3D structures^1–6^. However, these methods suffer from inefficient imaging and encounter numerous technical challenges during tissue slicing, labeling, imaging, and image stitching processes. Moreover, tissue slicing can cause structural deformation and significantly increase the difficulty of achieving accurate 3D reconstruction. Consequently, slice reconstruction techniques face difficulties in widespread application within biological research due to issues such as low imaging efficiency, challenging sample preprocessing procedures, and complex data reconstruction. In recent years, the emergence of tissue clearing techniques has facilitated uniform transparency throughout entire organs by mitigating light scattering and absorption problems encountered in large-scale tissue imaging processes while enabling intact whole organ and whole-body imaging^7^. Furthermore, ExM even enables nanoscale resolution enhancement of uniformly expanded tissue organs in three dimensions^8,9^. The advancement of biological tissue clearing technology effectively overcomes major barriers that impede the utilization of fluorescence microscopy for high-resolution 3D imaging of biological tissues. This breakthrough allows for efficient acquisition of three-dimensional structural information from biological tissues using state-of-the-art 3D fluorescence microscopy techniques^6,10–22^.

Tissue clearing techniques involve numerous methods that adhere to similar underlying principles. Based on these principles, the techniques can be categorized into hydrophobic, hydrophilic, and hydrogel methods. Taking uDISCO as an example, the hydrophobic approach involves three sequential steps: dehydration, delipidation, and refractive index matching. Notably, the dehydration process results in approximately a 50% reduction in sample volume which may impact delicate biological structures. Additionally, samples treated with hydrophobic clearing methods exhibit a refractive index around 1.56; consequently, achieving high-resolution imaging on processed samples is challenging due to refractive index mismatch with limited availability of high numerical aperture objectives. The samples prepared using the hydrophilic and hydrogel clearing methods maintain their original size or exhibit expansion while possessing a refractive index ranging from 1.33 to 1.52 making them suitable for high-resolution imaging applications.

**Figure 1:**
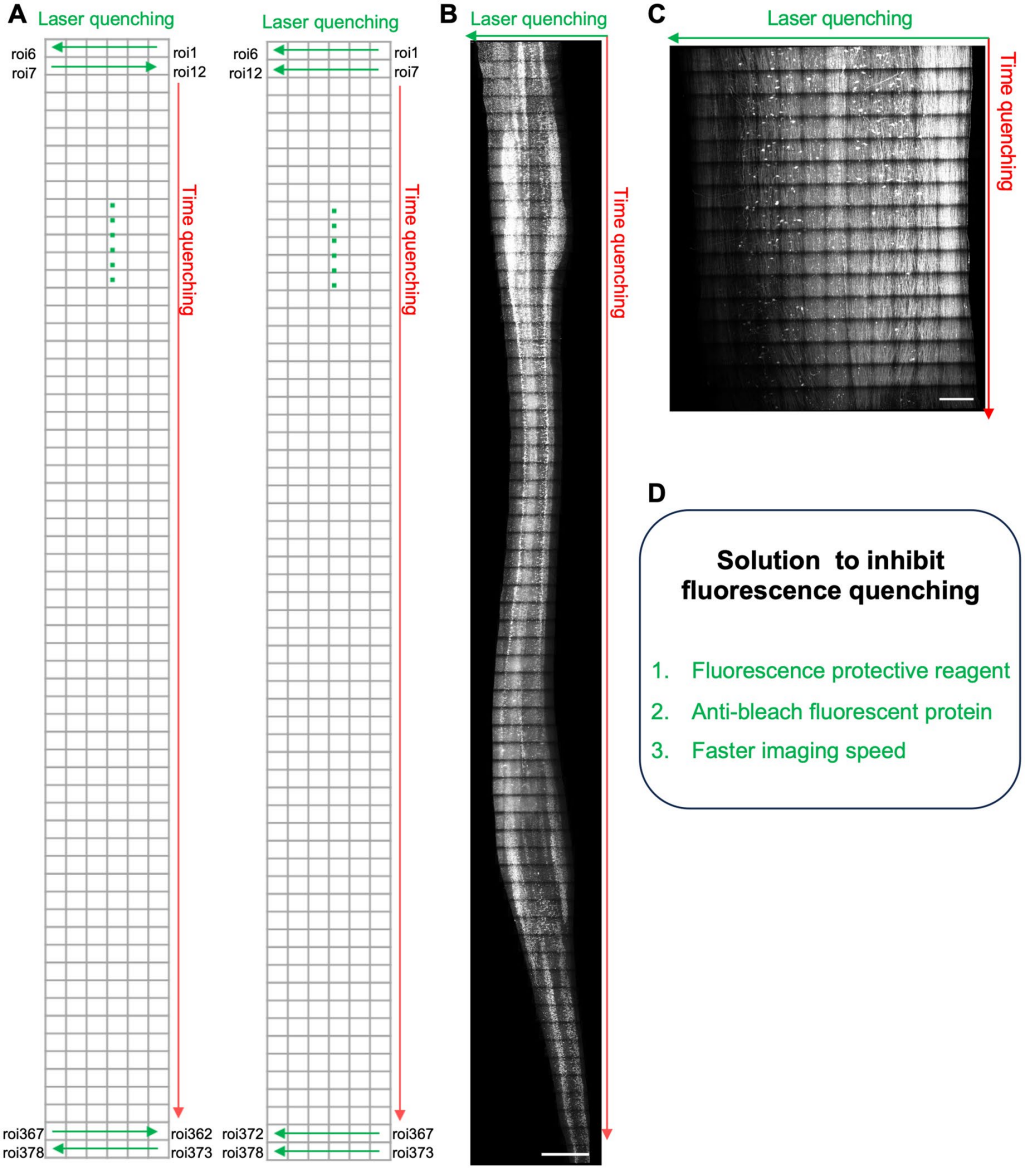
Challenges and potential solutions for subcellular high-resolution imaging of large tissues/whole organs. To perform subcellular high-resolution 3D imaging of cleared large tissues, it is essential to partition them into multiple sub-volumes, image them sequentially, register the images, and ultimately obtain a comprehensive 3D image. (**A**) For instance, in order to obtain subcellular high-resolution 3D images of the cleared spinal cord from Thy1-GFP mouse, tilling light-sheet microscopy (5X, 5 tiles, objective NA = 0.6) was employed for image acquisition. The target area was divided into a lattice of 6 by 63 regions of interest (ROIs), which was imaged row by row. (**B**) A time frame of approximately 7 days was required to complete single-channel fluorescence imaging of the cleared adult mouse spinal cord. The final composite 3D image was presented after performing image registration. (**C**) In order to obtain high-resolution 3D images of the expanded cervical segment of the spinal cord, sequential imaging was performed on a grid consisting of 13 rows and 16 columns of sub-volumes that are subsequently stitched together. Notably, our imaging results demonstrated a significant decrease in fluorescence intensity within areas imaged later compared to those captured initially. (**D**) Effective strategies were needed to mitigate fluorescence quenching during the imaging procedure. (Scale bar: 3000 μm).

Light sheet microscopy technology, characterized by its high speed, high resolution, and high signal-to-noise ratio, is highly suitable for three-dimensional imaging of transparent biological tissues^7,23–38^. By replacing physical slicing with optical sectioning, light sheet microscopy significantly enhances the speed and resolution of high-resolution three-dimensional fluorescence imaging of biological tissues^39^. Although exhibiting much less photobleaching and phototoxicity compared to wide-field fluorescence, confocal or multiphoton microscopes^40^,serious photobleaching issues can still arise as spatial resolution improves and tissue size increases. Two factors contribute to this issue: firstly, the sample volume is much larger than the thinnest waist length available in light sheets. During light sheet microscopy imaging, all fluorescent molecules along the illumination path are illuminated while only photons emitted from the waist region of the light sheet are collected and focused onto the detection camera. Due to limitations in camera pixels, imaging of an entire large sample in 3D is achieved by sequentially imaging adjacent subregions of the sample followed by image registration and merging. Consequently, a significant portion of these regions is bleached before actual imaging occurs (Fig. 1A). The extent of photobleaching can be estimated by comparing the excitation length with respect to the sample length. Clearly, a smaller ratio results in more severe photobleaching which hinders achieving higher spatial resolutions for a given size of sample or the maximum sample size that can be imaged at a desired spatial resolution. Another reason is that the number of fluorescent monomers per unit volume of a given sample remains constant. As spatial resolution increases, the field of view becomes smaller, resulting in a reduced number of available fluorescent monomers within the volume. To enhance the signal-to-noise ratio, it is necessary to extend the exposure time, which consequently prolongs the photobleaching duration in the imaged area. Apart from laser-induced photobleaching, high-resolution imaging of large biological samples often requires weeks or even longer periods, during which fluorescence similarly diminishes (Fig. 1B, C). To address this problem, three solutions can be implemented: (1) Selecting fluorescence protective reagents to prevent photobleaching of fluorescent proteins; (2) Choosing more stable fluorescent proteins with resistance to photobleaching; (3) Increasing the speed of imaging and reducing the impact of long-term imaging (Fig. 1D). In this study, we aim to screen suitable compounds to protect fluorescent proteins in transparent sample agents from quenching.

## Results

To address the issue of fluorescence protein quenching during prolonged imaging of cleared biological samples, we selected two structurally analogous compounds: Triethanolamine (Trie) and Ethylenediamine-*N*,*N*,*N*′,*N*′-tetra-2-propanol (EDTP). The protective effect on GFP was observed.

### Screening reagents to protect the fluorescence of fluorescent proteins

**Figure 2:**
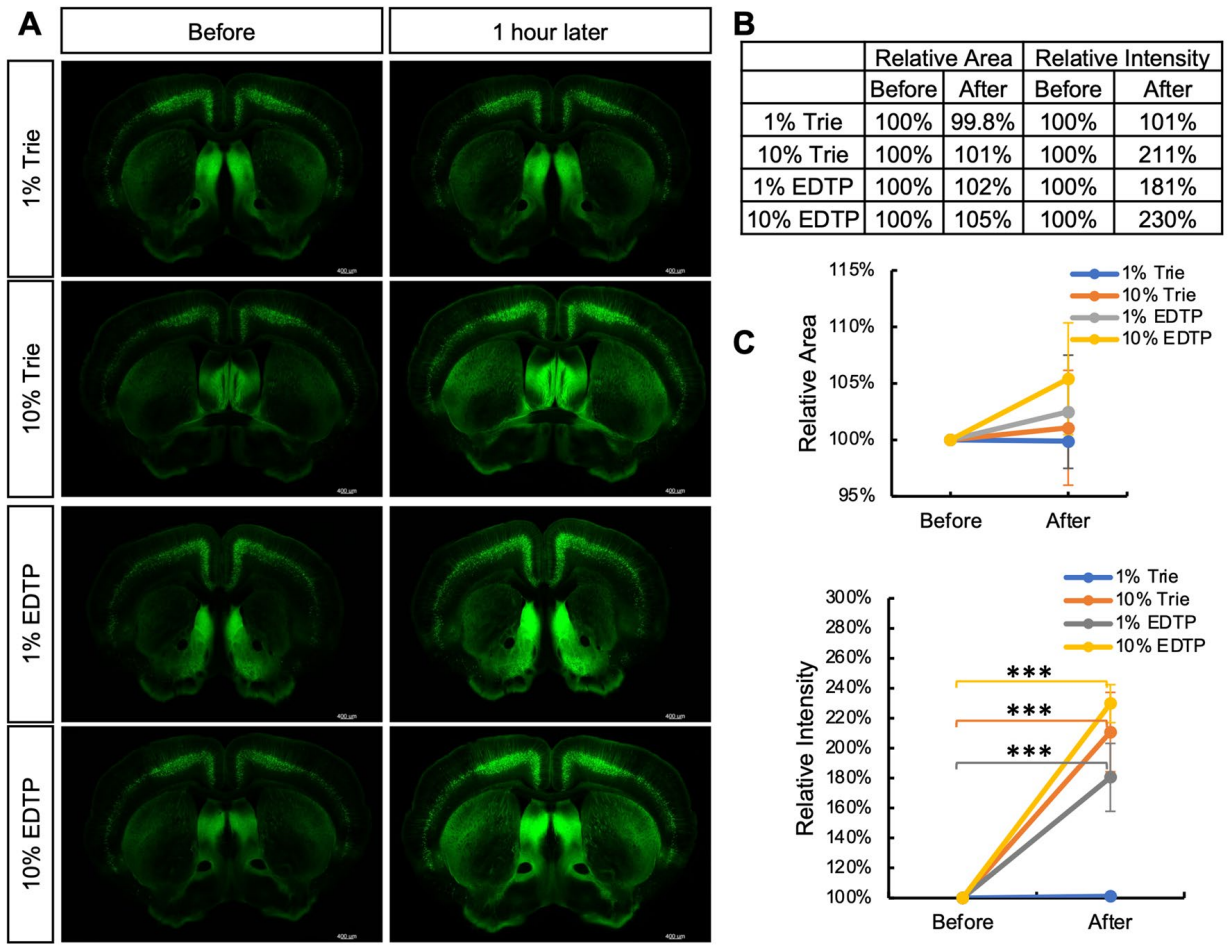
Fluorescence enhancement of different reagents on GFP in mouse brain slices. (**A**) Brain slices from adult Thy1-GFP mice with 200 μm thickness were imaged before and after incubation with various reagents for 1 h. (**B**, **C**) Changes in slice area and fluorescence intensity before and after treatment with different reagents were measured. The groups with the smallest effect on the brain slice size were 1% Trie, 10% Trie and 1% EDTP. It was worth noting that the fluorescence intensity increased significantly after treatment with 10% Trie and 1% EDTP (n > 4/group; Statistical analysis was performed using Student’s t test; ***p < 0.0001. Scale bar: 400 μm).

**Figure 3:**
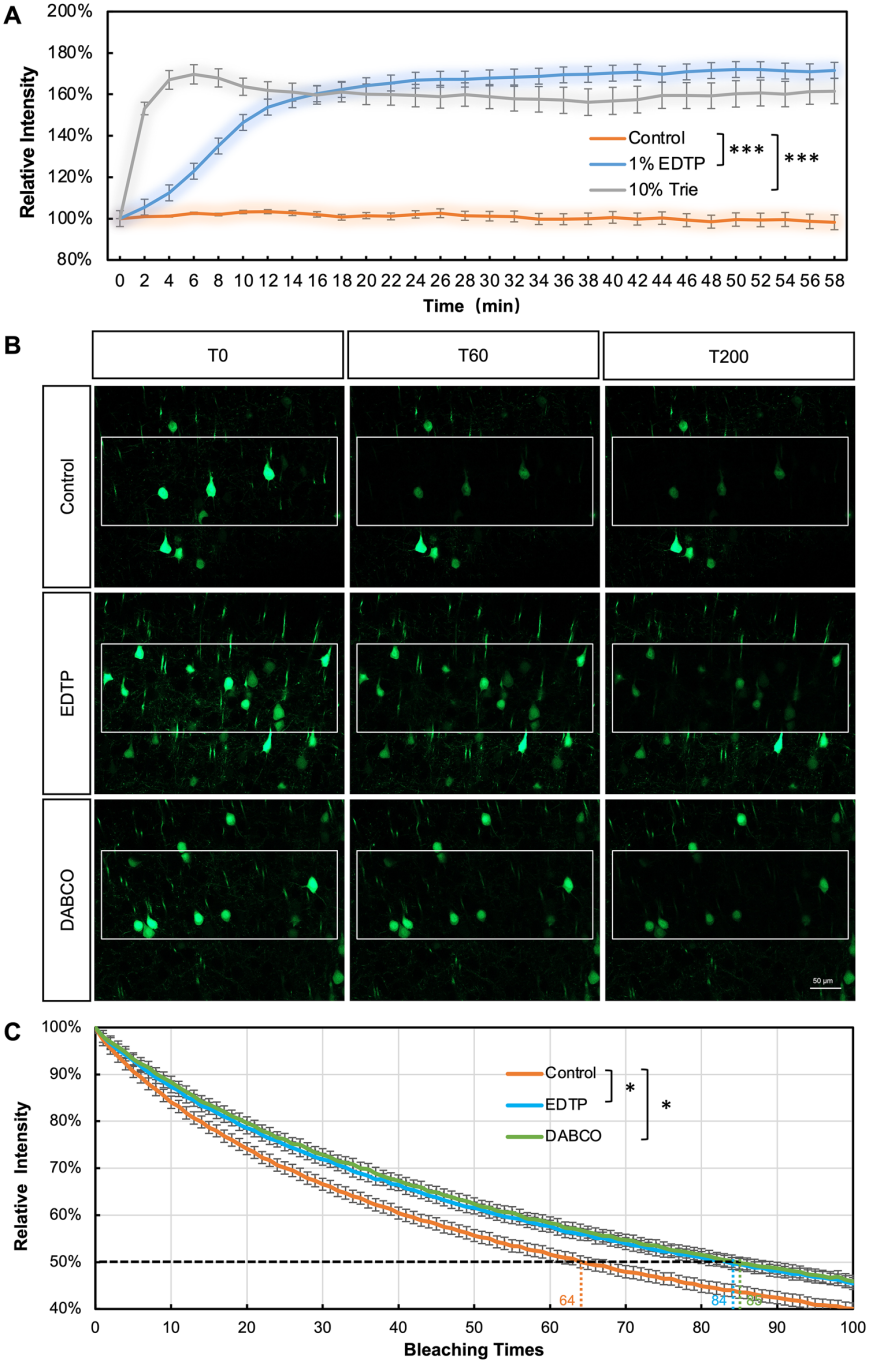
EDTP enhances the fluorescence intensity of GFP and resists quenching. (**A**) Real-time imaging of brain sections from adult 200 μm Thy1-GFP mice showed that incubation with 10% Trie or 1% EDTP rapidly enhanced GFP fluorescence intensity. Specifically, fluorescence intensity reached its peak after 6 min of treatment with 10% Trie, and then gradually decreased to a stable level. In contrast, GFP fluorescence intensity gently increased upon incubation with 1% EDTP until reaching its peak after approximately 30 min while remaining stable thereafter. Compared to 10% Trie, 1% EDTP improved GFP fluorescence intensity more consistently. (**B**, **C**) The quenching test was performed on the brain slices of Thy1-eGFP adult mice with 20% laser intensity. The half-life of 1% EDTP was similar to that of the classical anti-quenching agent 2.5% DABCO (T84 and T85, respectively), which was significantly higher than that of the control group (T64). (DABCO: Triethylenediamine) (n > 10/group; Statistical analysis was performed using one-way ANOVA; *P < 0.05, ***p < 0.0001. Scale bar: 50 μm).

First, the impact of these compounds on GFP in traditional vibratome sections was assessed. Inverted laser confocal microscopy was employed to visualize 200 μm slices from Thy1-GFP mouse brain, which were subsequently incubated in 0.01 M PBS containing 1% Trie, 10% Trie, 1% EDTP and 10% EDTP for one hour prior to reimaging (Fig. 2A). The analysis of alterations in the area of brain slices revealed that these compounds induce deformation in the slices (Fig. 2B, C), which was quantified by measuring the change in slice area pre- and post-incubation with each compound. Among them, groups with minimal impact on slice morphology: 1% Trie, 1% EDTP, and 10% Trie were Identified. These groups result in slice areas equivalent to approximately 99.8%, 101%, and 102% of their original size respectively. Incubation with 1% EDTP and 10% Trie led to an increase in GFP fluorescence intensity by approximately 181% and 211%, compared to its initial level. Subsequently, we performed real-time imaging to observe the dynamic process of GFP fluorescence enhancement (Fig. 3A). Brain slices from Thy1-GFP mice with a thickness of 200 μm were placed on an inverted fluorescence confocal microscope, and imaging was initiated upon addition of either 10% Trie or 1% EDTP. The results showed that both groups rapidly increased GFP fluorescence intensity. However, samples treated with 10% Trie reached their peak intensity after approximately 6 min before gradually declining to a stable level. In contrast, samples treated with 1% EDTP exhibited a gradual and sustained increase in fluorescence intensity for about 30 min. Notably, the enhancement of GFP fluorescence by 1% EDTP showed greater consistency and stability when compared to other conditions.

In order to evaluate the anti-photobleaching ability of EDTP, a commercially available anti-quenching agent known as DABCO was employed as a comparative standard. Thy1-GFP brain slices were incubated in PBS, 1% EDTP, and 2.5% DABCO respectively, and subjected to photobleaching using a 488 nm laser (20% laser intensity) for a total of 200 cycles (Fig. 3B). The results showed that in the control group, fluorescence intensity decreased to 50% after undergoing 64 bleaching cycles, while samples incubated with EDTP and DABCO reached this level at approximately 84 and 85 bleaching cycles respectively (Fig. 3C). This means that 1% EDTP possesses comparable anti-photobleaching ability as compared to 2.5% DABCO. Overall, it can be concluded that by incubation with 1% EDTP, GFP fluorescence intensity can be enhanced while simultaneously safeguarding against photobleaching without significantly impacting tissue morphology.

### EDTP can enhance the intensity of GFP and provide long-term protection in cleared tissues.

**Figure 4:**
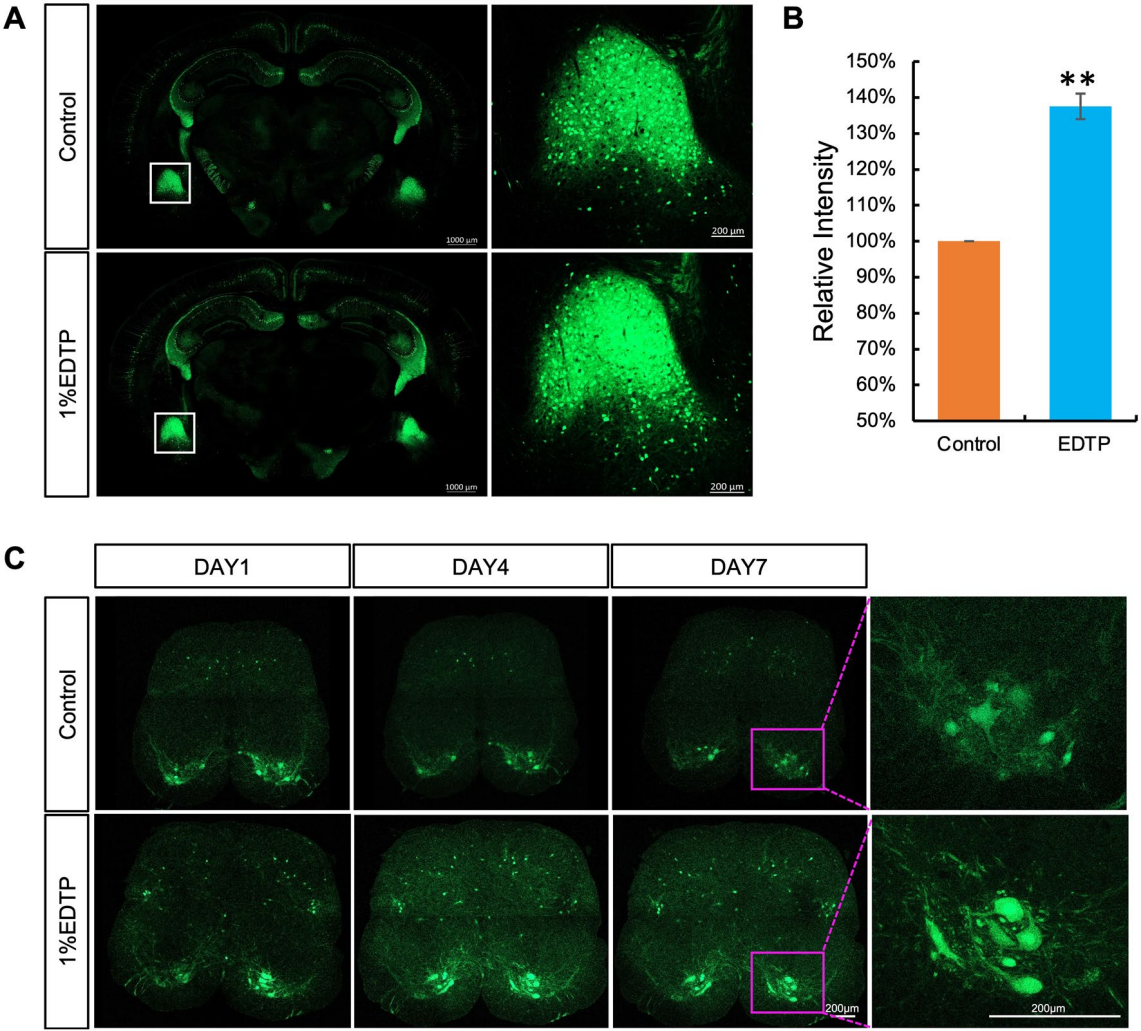
EDTP enhances the fluorescence intensity of GFP in tissue clearing samples and provides long-term protection. (**A**) Brain slices, 200 μm thick, from adult Thy1-GFP mice underwent the standard tissue clearing procedure involving lipid removal and refractive index (RI) matching before being imaged on an inverted fluorescence microscope. Subsequently, they were incubated with a RI matching solution containing 1% (w/w) EDTP for 1 h prior to reimaging. (**B**) It was observed that incubation with 1% (w/w) EDTP resulted in a significant increase of 138% in the fluorescence intensity of GFP (n > 3/group; **p < 0.01). (**C**) Spinal cord slices, 200 μm thick, obtained from adult ChAT-GFP mice were subjected to lipid removal and matched with RI matching solution containing 1% (w/w) EDTP. Subsequently, the samples were mounted using a gelling solution composed of 2% agarose and 1% EDTP in RI matching solution. Imaging was conducted on the day of sample preparation as well as on day 4 and day 7. The results demonstrated that compared to the control group, the fluorescence signal of GFP in the EDTP-treated samples exhibited prolonged maintenance.

To investigate the protective effects of EDTP on GFP in cleared samples, we performed a lipid removal and refractive index matching protocol on 200 μm Thy1-GFP adult mouse brain slices. We imaged the slices and then incubated them in a refractive index matching solution containing 1% EDTP for one hour before imaging again. By comparing the GFP fluorescence intensity before and after EDTP incubation, we observed a significant increase in GFP fluorescence intensity in cleared brain slices to 138% of its original level (Fig. 4A and, B). To assess whether EDTP can provide long-term protection against bleaching caused by environmental factors in cleared tissues, we subjected 200 μm thick ChAT-GFP adult mouse spinal cord slices to lipid removal and matched them with a refractive index matching solution containing 1% EDTP. The cleared slices were then spread out on the bottom of a confocal dish and covered with 2% agarose at 4℃. After agarose gelation, an imaging solution was applied to simulate the conditions during imaging of transparent samples. Imaging was performed on the day of sample preparation as well as on the fourth and seventh days thereafter (Fig. 4C). Compared to the control group, samples treated with EDTP exhibited prolonged preservation of GFP fluorescence signal over time.

**Figure 5:**
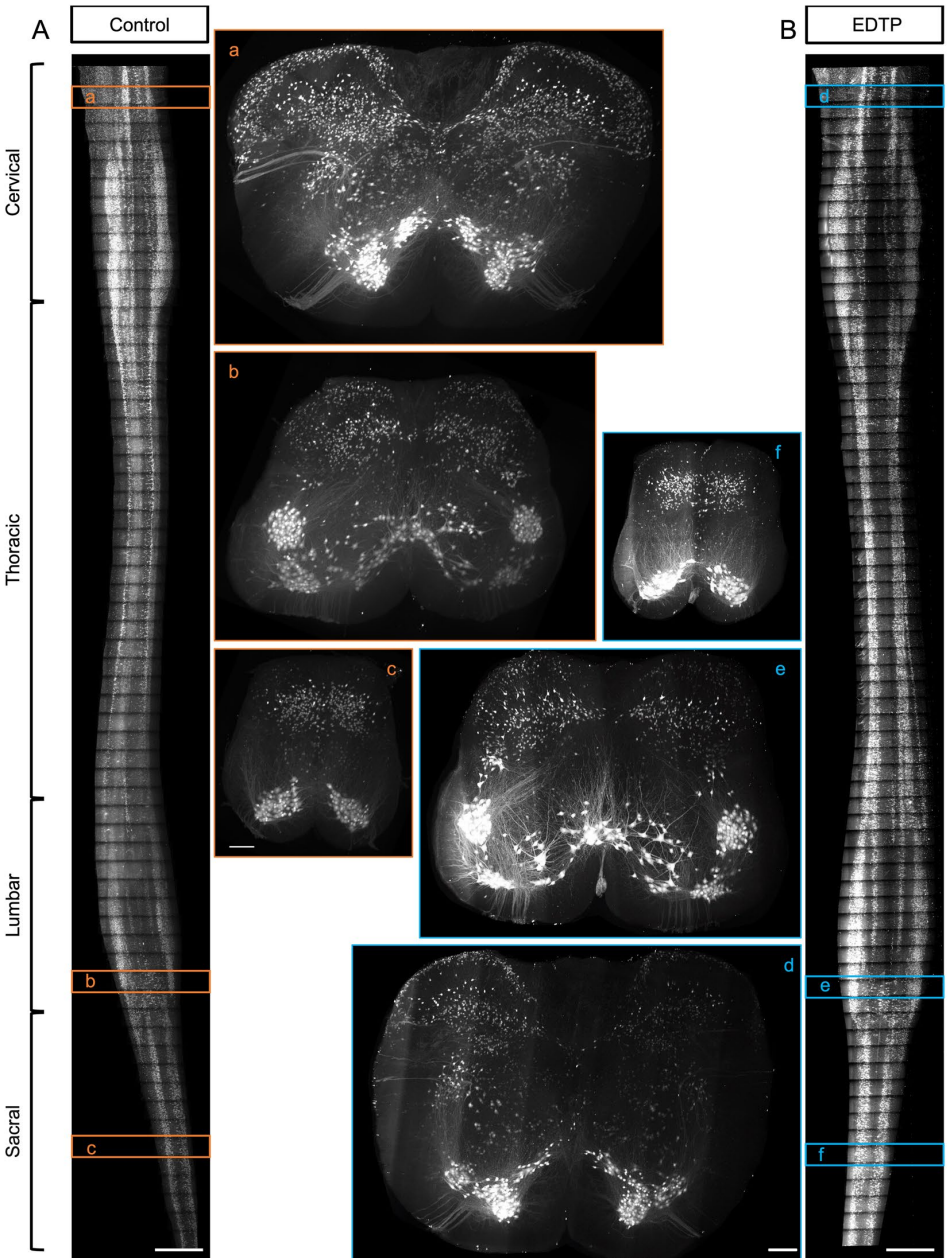
EDTP provides long-term protection for GFP in cleared sample. (**A** and **B**) To achieve high-resolution imaging of the intact mice spinal cord (ChAT-GFP), it was necessary to divide the spinal cord into approximately 6 by 63 regions of interest (ROI) and captured images row by row over a period of about 7 days (**A**, **B**) represented the maximum intensity projection (MIP) in the XY direction of the cleared spinal cord, (a–c) are the MIPs in the XZ direction for region A, (d–f) were the MIPs in the XZ direction of the region shown in B. (**A**) The result showed a significant decrease in fluorescence intensity for the regions imaged later (c) compared to those imaged earlier (a). (**B**) Samples treated with EDTP exhibit consistent fluorescence intensity throughout the entire week-long imaging process (d–e). (Scale bar: A, B is 3000 μm; a-f is 200 μm).

Then we analyzed the preservation of GFP fluorescence signal over time between samples treated with EDTP The adult Chat-GFP mouse spinal cords were subjected to lipid removal, refractive index matching, and gel embedding for 3D imaging using a tilling light sheet microscope as previously described^41^. In the experimental group, 1% EDTP was incorporated into the refractive index matching solution and the corresponding gel solution. NA 0.6 Objective (5X magnification, 5 tiles) was employed to capture whole-spinal cord images in gel-embedded samples as depicted in Fig. 1A. In the control group, noticeable fluorescence attenuation occurred when imaging from cervical to sacral segments of the spinal cord (Fig. 5A–C). However, in the sample treated with 1% EDTP, no significant alteration in GFP fluorescence was observed (Fig. 5D–F), indicating that EDTP exerted a substantial protective effect on GFP fluorescence during cleared whole-spinal cord imaging over a 7-day period. Following this step, we immersed the samples in the imaging solution and stored them at room temperature under light protection. After 6 weeks elapsed, we re-imaged the region shown in Supplementary Fig. 1A at the cervical segment and noted no obvious change of GFP fluorescence compared to before (Supplementary Fig. 1B). This result suggested that EDTP provide long-term protective effects on fluorescent proteins within cleared samples.

### EDTP preserves the fluorescence intensity of GFP in expanded tissues

**Figure 6:**
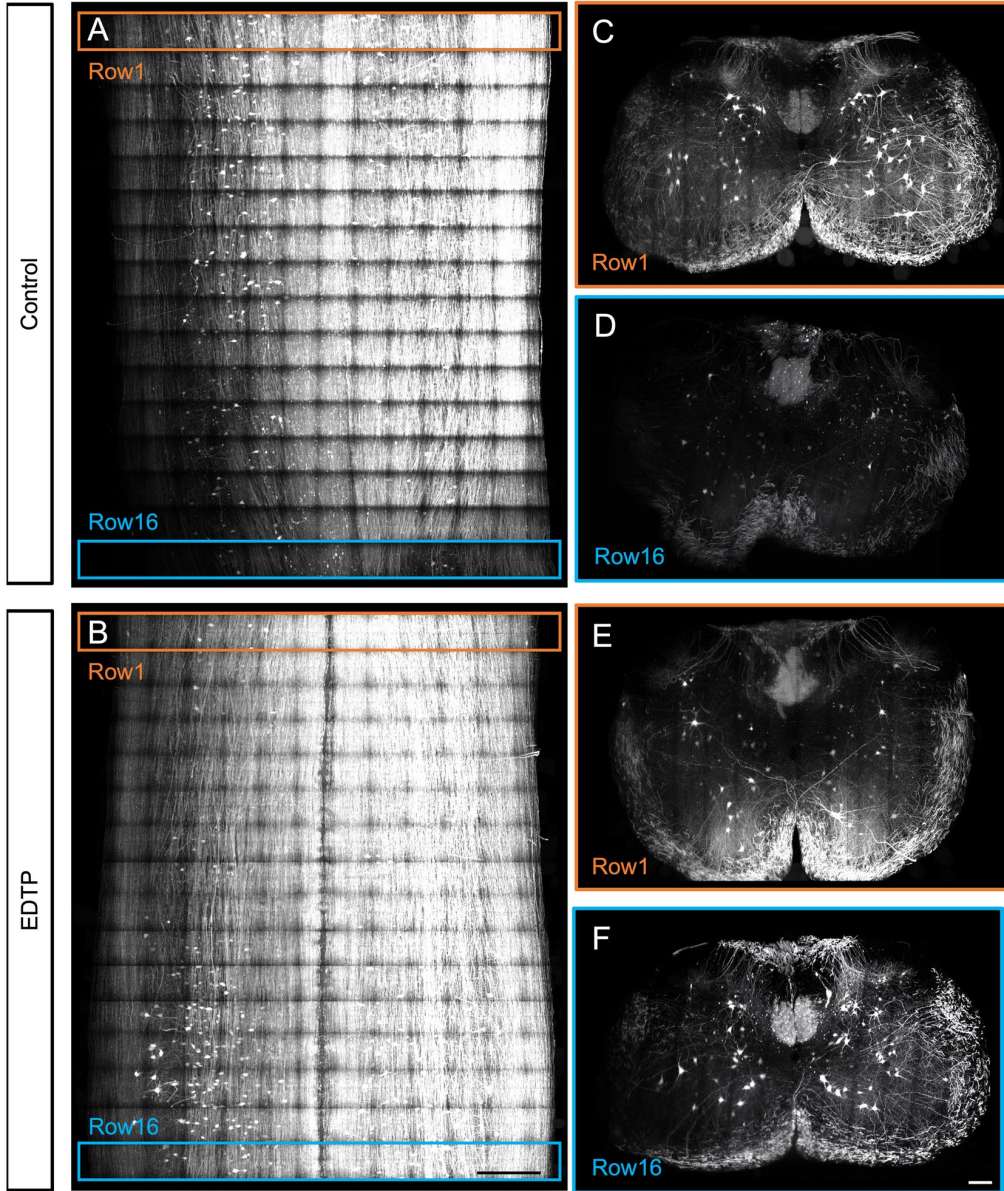
EDTP provided long-term protection for GFP in expanded sample. Expanded samples, derived from the cervical spinal cord of ChAT-GFP mice using CMAP method (expanded approximately fourfold), were imaged at high resolution with a 0.6NA objective lens. The spinal cord was divided into approximately 13 by 16 ROIs, and imaging was conducted row by row over a period of about 4 days. (**A**, **B**) represented the MIP in the XY direction of the expanded spinal cord, (**C** and **D**) are the MIPs in the XZ direction for region A, (**E** and **F**) were the MIPs in the XZ direction of the region shown in B. The data revealed a significant decrease in fluorescence intensity in later-imaged areas (**D**) compared to earlier-imaged areas (**C**). However, the fluorescence intensity remained stable throughout the imaging process in samples treated with EDTP (**E**, **F**). (Scale bar: 300 μm).

Similarly, a significant protective effect on GFP in samples prepared using the cMAP method for expansion microscopy was observed when 1% (w/w) EDTP was added, as shown in Fig. 6. Adult thy1-GFP mouse spinal cord cervical segments were expanded and imaged at high resolution. The control group used deionized water during sample expansion and imaging, while the test group employed deionized water containing 1% EDTP for sample expansion and imaging. Following sample preparation using the cMAP method, the cervical spinal cord segment of adult Thy1-GFP mice exhibited approximately fourfold unidirectional expansion compared to its original size. Tilling light-sheet microscopy (4X, 3 tiles, objective NA = 0.8) was used for the tissue imaging. During high-resolution imaging, each row consisted of 13 sub-volumes. By the time the 16th row was captured (imaging on day 3, Fig. 6A, C), a significant reduction in GFP fluorescence intensity was observed compared to the first row (imaging on day 1, Fig. 6A, D). In contrast, no apparent changes were detected in GFP fluorescence within the experimental group indicating that EDTP exerts a long-lasting protective effect on GFP in samples prepared using the cMAP method.

## Discussion

The integration of light sheet microscopy with tissue clearing and tissue expansion techniques obviates the need for physical tissue sectioning, rendering it a highly suitable approach for investigating intricate details within intact biological samples. Subcellular high-resolution fluorescence imaging of large tissue samples, such as the mouse spinal cord, often requires a substantial amount of time to complete. Imaging a single channel of fluorescence in the transparent adult mouse spinal cord typically takes approximately 7 days. However, due to significant decay in the fluorescence signal of the fluorescent protein over time, it is imperative to develop methods for long-term preservation of this signal. These challenges can be addressed by substituting more stable fluorescent proteins and fluorophores that are resistant to quenching, incorporating fluorescent protectants, or implementing novel 3D imaging strategies.

In this study, our objective is to identify compounds that can safeguard the fluorescence intensity of fluorescent proteins during the cleared or expended sample preparation process and sustain it for an extended duration without compromising tissue morphology. We have chosen structurally analogous counterparts, namely EDTP, and Triethanolamine (Trie), which have been employed in diverse hydrophilic-based clearing techniques. EDTP have been reported to possess decolorizing properties and are used in the decolorization and dilipidation step of hydrophilic clearing methods^7,31^. Triethanolamine (Trie) is used for refractive index matching^7^. Additionally, EDTP has been reported to exhibit antioxidant properties by scavenging free radicals, thereby protecting fluorescent proteins in hydrophobic transparency method FDISCO + ^42^. Initially, we investigated the impact of varying concentrations of EDTP and triethanolamine (Trie) on the morphology of normal brain slices and the fluorescence intensity of endogenous fluorescent protein GFP. In the upper panel of Fig. 2C, we examined the area change in tissue samples before and after drug treatment. Our statistical analysis revealed no significant differences in the area measurements pre- and post-treatment. This lack of significant change may stem from the inherent structural and compositional variations among brain slices, which could introduce variability within each group. Given the consideration of morphology maintain, we selected the groups that exhibited a minimal area change coupled with a significant increase in fluorescence for further investigation. This approach ensures that our subsequent studies focus on the most consistent and reliable outcomes, providing a robust foundation for our research. Subsequently, their dynamic influence on fluorescence intensity was observed through real-time imaging, while their resistance to photobleaching was evaluated. Ultimately, 1% EDTP was selected to preserve GFP fluorescence in cleared and expanded samples for an extended period.

In our quest to elucidate the protective mechanism of EDTP on GFP, we commenced our investigation by assessing the presence of free radicals within our experimental conditions. Our results revealed no detectable presence of free radicals (data not show), although this could potentially be attributed to limitations in the sensitivity of our detection method. Furthermore, a panel of established antioxidants, including rutin, quercetin, vitamin C, glutathione, uric acid, α-lipoic acid, and epigallocatechin gallate, failed to replicate the unique effect observed with EDTP (data not show). This led us to conclude that EDTP enhances the brightness of GFP and offers enduring protection during sample preparation and imaging processes, independent of free radical scavenging. Further investigation is warranted to investigate whether EDTP can confer comparable protection to all fluorescent proteins like GFP and elucidate its underlying mechanism of action. EDTP can be incorporated into conventional fluorescent mounting medium, hydrophilic clearing reagents, and the hydro-gel expansion method to augment the fluorescence intensity of fluorescent proteins while ensuring long-term preservation of the fluorescence signal. This will aid in obtaining comprehensive tissue structures at the subcellular level and advancing research in fields such as neural connectivity mapping and tumor microenvironment analysis.

## Methods

### Animals

All animal work was performed in accordance with protocols approved by the Institutional Animal Care and Use Committee of the Westlake University (Approval No: 19-035-GL), Hangzhou, China. The study was approved by the Institutional Animal Care and Use Committee of the Westlake University (Approval No: 19-035-GL), Hangzhou, China. The part of our study involving animal work is reported in accordance with ARRIVE guidelines ([https://arriveguidelines.org) .] https://arriveguidelines.org ). The Thy1-GFP (Tg(Thy1-eGFP)MJrs/J, Stock No: 007788) strain was generously provided by Dr. Yuqiang Ding from Fudan University, while the ChAT-GFP (B6.Cg-Tg(RP23-268L19-EGFP)2Mik/J, Stock No: 007902) strain was kindly provided by Dr. Liang Wang from Zhejiang University. All mice were housed in a controlled environment with a 12-h light–dark cycle.

### Tissue collection

Adult mice (about P60) were deeply anesthetized by intraperitoneal injection of pentobarbital (approx. 150 mg/kg of body weight) and perfused with heparinized 0.01 M PBS (10 units/ml of Heparin) at 10 ml/min for 5–10 min until the blood was washed out. Followed by perfusion with 4% PFA (pH 7.4) for another 5–10 min. Brains and spinal cords were harvested following a previously reported protocol^43^ and post-fixed in cold 4% PFA overnight with gentle shaking. After rinsing with PBS, tissues were sliced on a vibratome (Leica VT1200S) or processed by tissue clearing and expansion.

### Fluorescence microscopy imaging

Tissue slices were laid on the bottom of confocal dishes and imaged by inverted laser confocal microscope (Zeiss LSM880,Zeiss LSM980). The sections were subjected to fluorescence imaging before and after incubation with different drugs using 488 nm laser with 0.1% laser energy (equivalent to 0.002 W/cm^2^). Real-time effects of drug incubation on fluorescent proteins were imaged in time series or bleaching modes. Time series imaging was performed with a rate of 1 frame every 2 min. In the bleaching mode, 488 nm laser with 20% laser energy (0.4 W/cm^2^) was used for fluorescence quenching and the same area was quenched 200 times. The original image was collected before quenching, and then the image was captured one at a time after bleaching.

### Tissue clearing and tissue expansion

Tissue clearing and tissue expansion were performed as previous reported^41,44^. For tissue clearing, the sample was first incubated in the delipidation solution (15 wt/wt% urea, 10% wt/wt% *N*-butyldiethanolamine, 10 wt/wt% Triton X-100 and 65 wt/wt% ddH_2_O) at 37 °C for 3–5 days and then transferred to the RI matching solution (25 wt/wt% urea, 22.5 wt/wt% sucrose, 22.5 wt/wt% antipyrine, 10 wt/wt% triethanolamine) at 25 °C until the sample was transparent. The delipidation solution and RI matching solution should be changed daily. For tissue expansion, delipidated samples were first washed with PBS, and then incubated in monomer solution (30% Acrylamide, 0.075% *N*,*N*-Dimethylacrylamide, 10% Sodium acrylate, 0.5% 2,2′-Azobis dihydrochloride in 0.01 M PBS) at 4 °C for 2 days. After that, the monomers were induced to polymerize into hydrogels, and the expanded tissues were obtained after full absorption of DI water.

### High resolution 3D imaging of large tissue

The workflow for image acquisition, processing, registration, and merging was described in detail in our previous publications^45,46^. Adjust the microscope settings to achieve the desired level of magnification and reposition the focus onto the excitation beam. Then adjust the tiling position and intensity profile of the excitation light sheet. Generate and load phase maps for imaging conditions, capture images from various sub-volumes, reconstruct raw images acquired in each sub-volume into high-resolution image stacks. Finally, obtain a 3D image of the entire sample volume by registering and merging reconstructed 3D images of all sub-volumes using Amira software.

### Imaging analysis and statical analysis

Zeiss blue was used to measure fluorescence intensity. The data were presented as the mean ± standard error (SE) of at least three independent experiments. Multiple group comparisons were conducted using one-way analysis of variance (ANOVA), and two-group comparisons were analyzed using Student’s t-tests. Excel was used to analyze and plot. A significance level of *P < 0.05 was considered statistically significant.

### Supplementary Information

Supplementary Figure S1.

## Data Availability

The data used to support the findings of this study are available from the corresponding author upon request.
