# Supplementary material for: EDTP enhances and protects the fluorescent signal of GFP in cleared and expanded tissues
Source: Sci Rep. 2024 Jul 3;14:15279. doi: 10.1038/s41598-024-66398-y (PMC11222453; doi:10.1038/s41598-024-66398-y)
Supplement: Supplementary file 1 — Supplementary Figure S1. [file 41598_2024_66398_MOESM1_ESM.pdf]

Supplementary figures

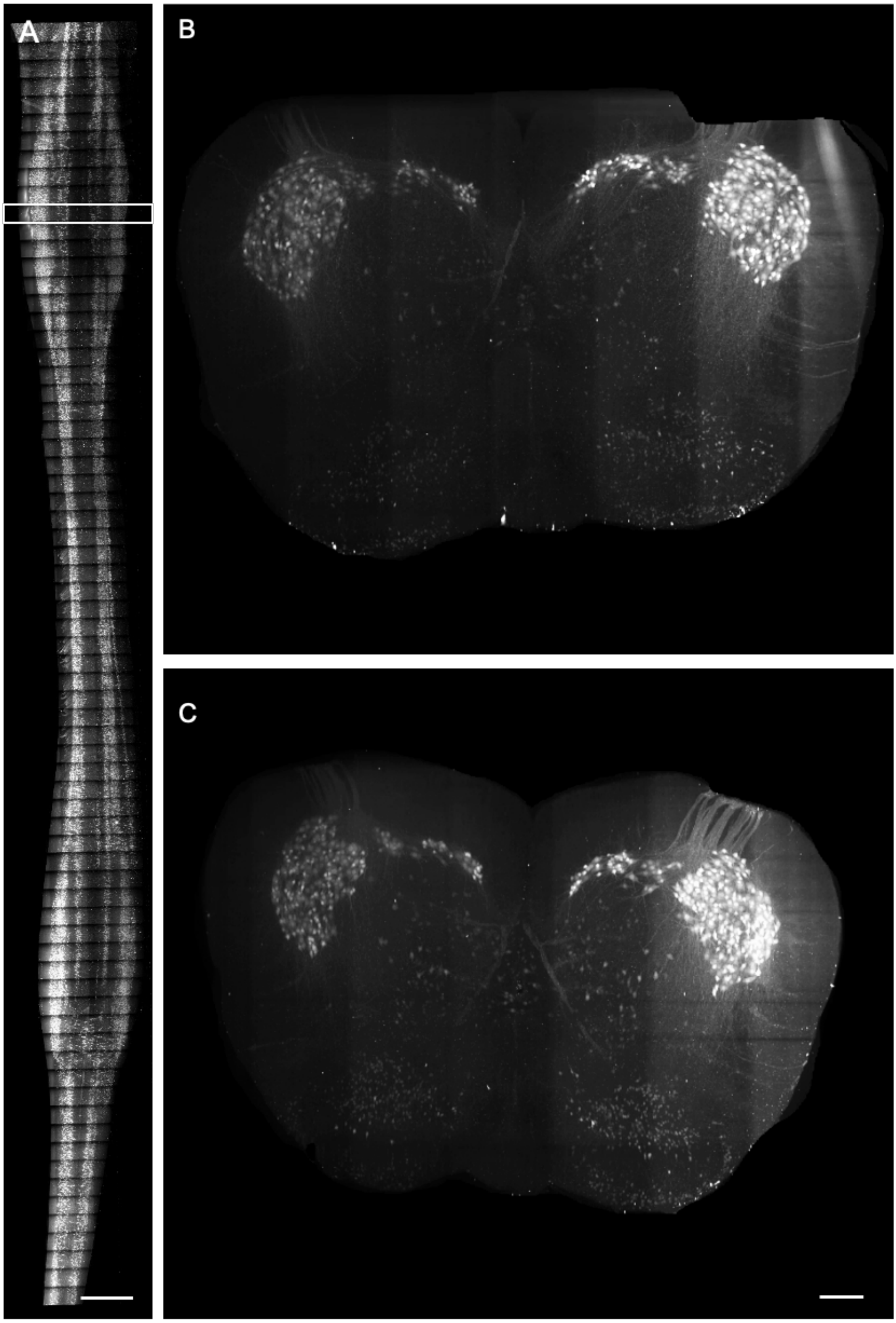

S1 FIG. EDTP has a long-term protective effect on fluorescent proteins in cleared samples. Following the imaging of the entire spinal cord from ChAT-GFP mouse, the samples were immersed in the imaging solution and stored at room temperature away from light (A). After 6 weeks, re-imaging of the cervical segment region shown in Figure A revealed that the fluorescence intensity of GFP did not decay compared to that of the same region before 6 weeks (B, C), indicating a sustained protective effect of EDTP on fluorescent proteins in transparent samples. (Scale bar: 300μm).
